# Data supporting the regulation of FOXC2 in podocyte dysfunction

**DOI:** 10.1016/j.dib.2015.12.051

**Published:** 2016-01-07

**Authors:** Neeta Datta, Sonja Lindfors, Naoyuki Miura, Moin A. Saleem, Sanna Lehtonen

**Affiliations:** a Department of Pathology, University of Helsinki, 00290 Helsinki, Finland; b Department of Biochemistry, Hamamatsu University School of Medicine, Hamamatsu 431-3192, Japan; c Academic and Children’s Renal Unit, Dorothy Hodgkin Building, Bristol BS1, United Kingdom

## Abstract

This data article shows the expression levels of specific podocyte injury markers and podocyte slit diaphragm protein nephrin in obese and lean Zucker rat glomeruli. It also contains information on the effect of the overexpression of transcription factor FOXC2 on the ratio of F- and G-actin and the expression level of ZO-1 in differentiated human podocytes. The article also shows data on the effect of treatments of differentiated podocytes with various factors associated with obesity and diabetes on the expression level of FOXC2. The detailed interpretation of these data and other aspects of podocyte injury mediated by upregulation of FOXC2 can be found in “Overexpression of transcription factor FOXC2 in cultured human podocytes upregulates injury markers and increases motility [1].

**Specifications table**

**Table:** 

Subject area	*Nephrology*
More specific subject area	*Obesity-related kidney injury*
Type of data	*Table, figures, description of accompanying methods*
How data was acquired	*Scanning with Odyssey Infrared Imager followed by quantification with the Odyssey software (LI-COR, Lincoln, NE, USA), performing quantitative RT-PCR with an iCyclerIQ*® *(BIO-RAD, Hercules, CA, USA).*
Data format	*Analyzed*
Experimental factors	*N/A*
Experimental features	*In vitro-treatment of cultured podocytes with various factors, quantitative Western blotting, quantitative RT-PCR, definition of F-actin/G-actin ratio*
Data source location	*N/A*
Data accessibility	*Within this article*

## Value of the data

• The obese Zucker rats show a trend towards upregulation of podocyte injury markers in glomeruli.
• The obese Zucker rats with the highest level of proteinuria express least nephrin in glomeruli.
• Overexpression of FOXC2 in differentiated human podocytes *in vitro* does not change the F-actin/G-actin ratio, or the expression level of the tight junction protein ZO-1.
• Several obesity and diabetes-associated factors were found not to upregulate FOXC2 in differentiated human podocytes.

## 1 Data

**Fig. 1:**
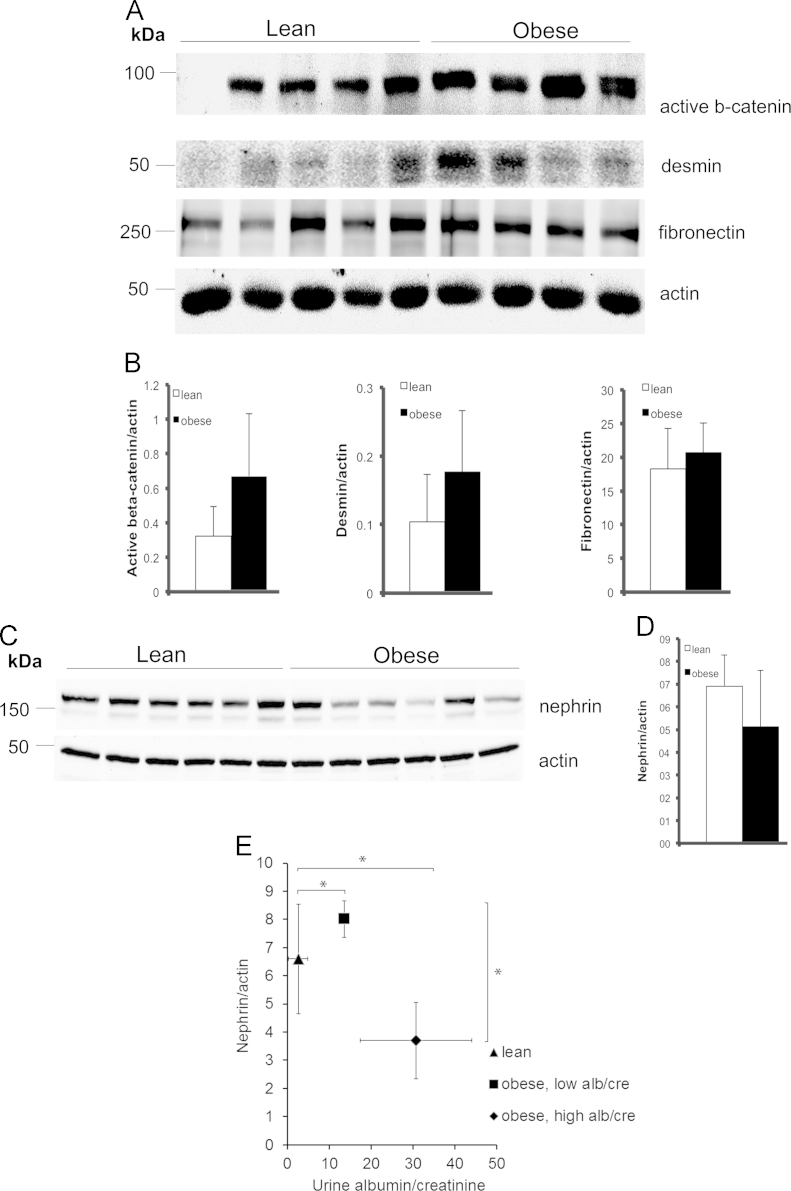
Immunoblotting reveals an increasing trend of key podocyte injury markers and decreasing nephrin expression in obese Zucker rat glomerular lysates. (A) 75 μg of glomerular lysates were separated by SDS-PAGE and immunoblotted for active β-catenin, desmin and fibronectin. Actin was used as the loading control. (B) Quantification shows an increasing trend in the expression of active beta-catenin, desmin and fibronectin in the glomeruli of the obese Zucker rats compared to the lean controls (*n*=5 for lean & *n*=4 for obese). (C, D) Immunoblotting for nephrin and subsequent quantification shows a decreasing trend in the expression of nephrin in the glomeruli of the obese Zucker rats compared to the lean controls. (*n*=6 for lean & *n*=6 for obese). None of the changes reached statistical significance because of high individual variation. (E) Comparison of urine albumin/creatinine ratio to nephrin/actin ratio (C) in Zucker lean and obese rats reveals that the obese rats with highest albuminuria (>15 mg/mg) express less nephrin in the glomeruli than the obese rats with low albuminuria (<15 mg/mg). Data are mean±SD (*n*=3 for lean, *n*=2 for obese with low alb/cre, *n*=4 for obese with high alb/cre). *≤0.05.

**Fig. 2:**
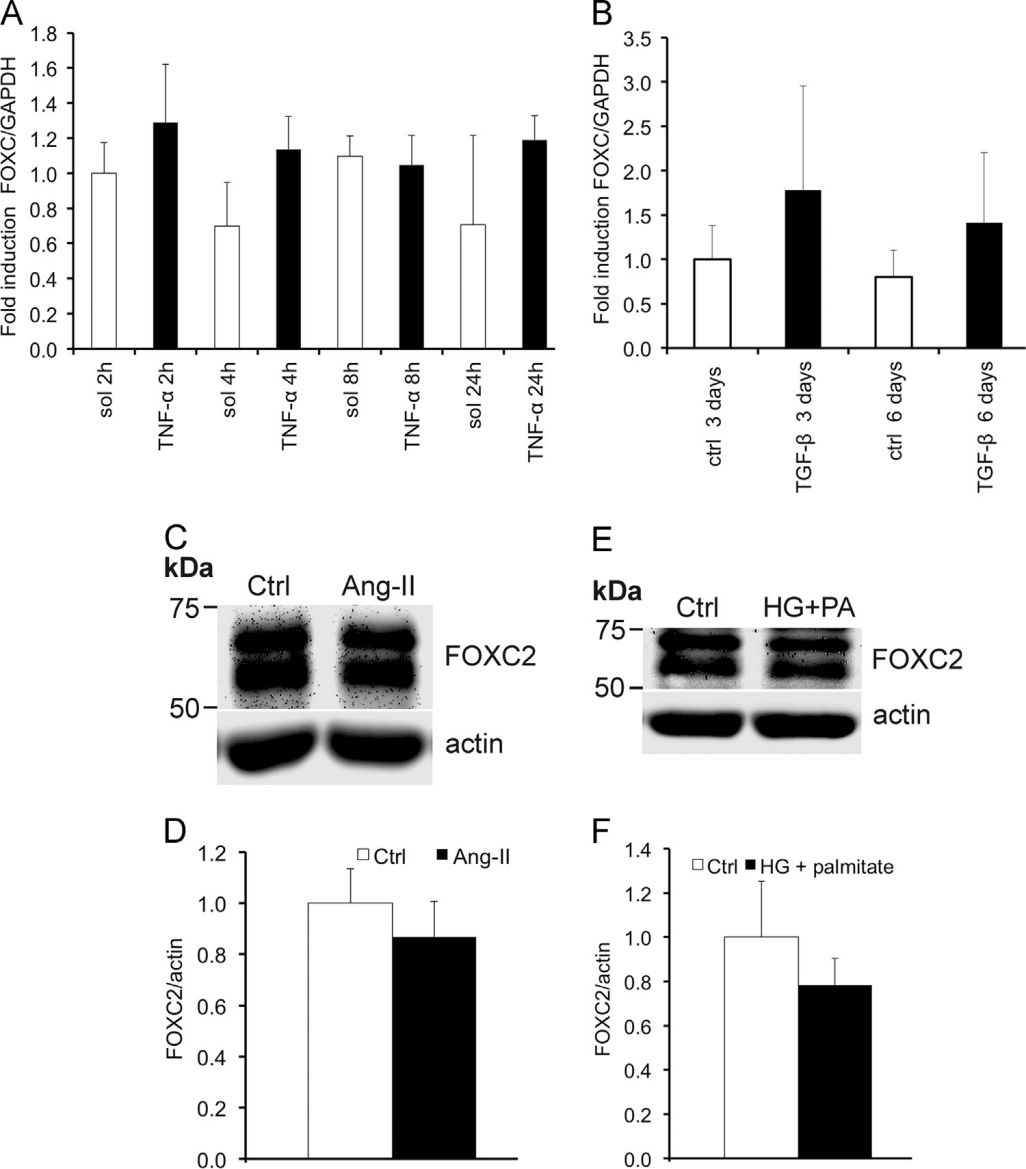
FOXC2 is not upregulated by TNF-α, TGF-β, angiotensin II (Ang II) or high glucose and palmitate treatment**.** (A and B) FOXC2 mRNAs were measured by quantitative RT-PCR and normalized to GAPDH mRNA using the comparative Ct method (DDCt) after treatment with 10 ng/ml TNF-α for 2–24 h (A) or 4 ng/ml TGF-β (B) for 3 or 6 days during the last days of differentiation. The values represent means and range of 3 measurements from two individual experiments. The experiments were repeated three times with similar results showing no significant change in the expression of FOXC2 mRNA in the treated cells compared to controls. (C and D) Immunoblotting and quantification of FOXC2 in differentiated podocytes treated with 1 μM angiotensin II for 24 h shows no difference in FOXC2 expression between the control and the treated cells. (E and F) Immunoblotting and quantification of FOXC2 in differentiated podocytes treated simultaneously with 20 mM glucose and 200 μM palmitate for the last 7 days of the differentiation period reveals no change in the expression of FOXC2 in the treated cells. Actin is included as the loading control. All data are mean±SEM (*n*=3 experiments).

**Fig. 3:**
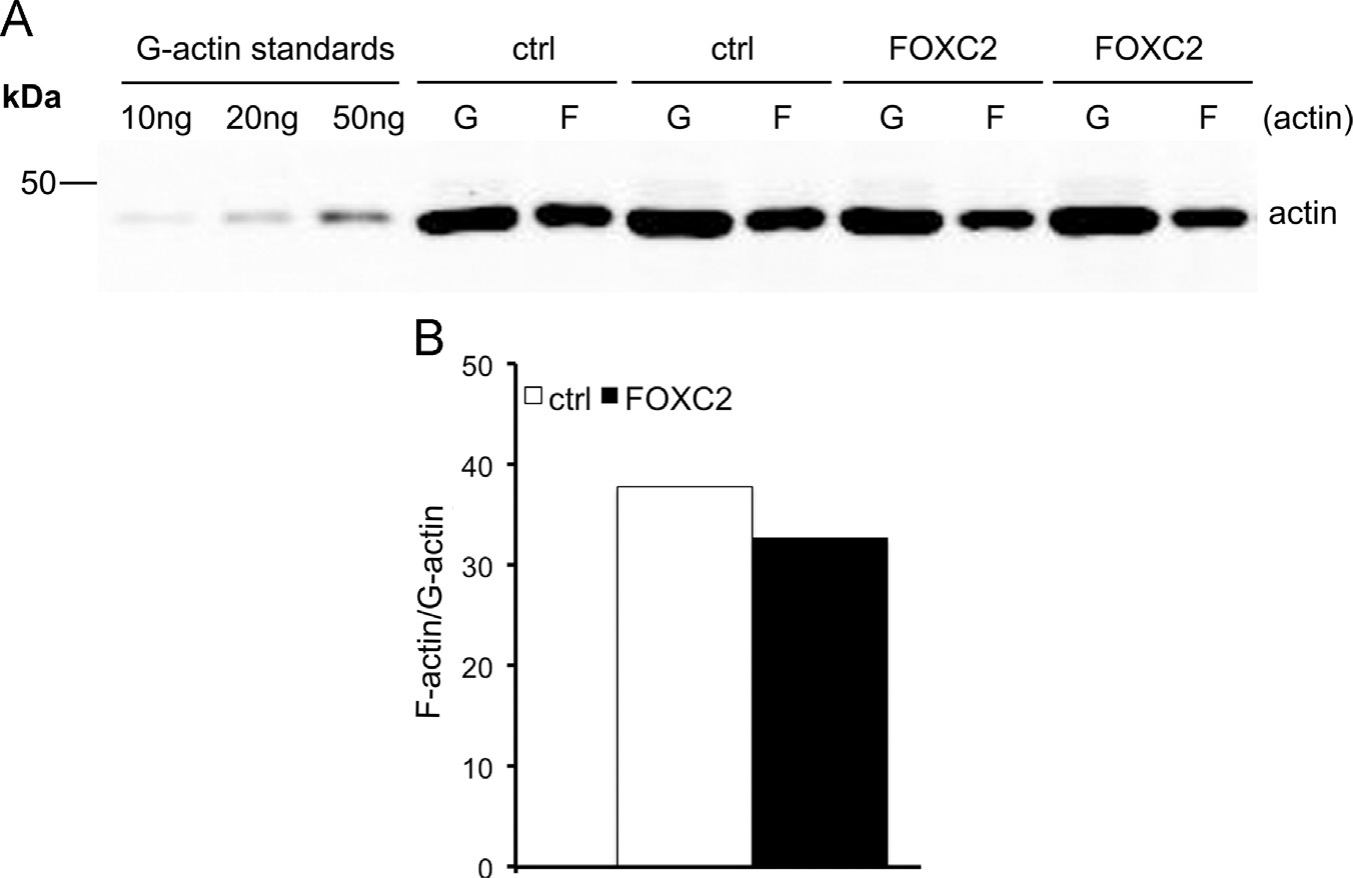
FOXC2 overexpression does not change the ratio between filamentous (F) and globular (G) actin**.** (A) Immunoblotting for actin (F- and G-actin) in differentiated podocytes overexpressing FOXC2 or an empty vector (ctrl). Cells were lysed and processed into supernatant (F-actin) and pellet (G-actin) fractions and immunoblotted for actin. (B) Densitometry reveals no difference in the F-actin/G-actin ratio between the control and FOXC2 overexpressing cells. Data are mean±SEM (*n*=3 experiments).

**Fig. 4:**
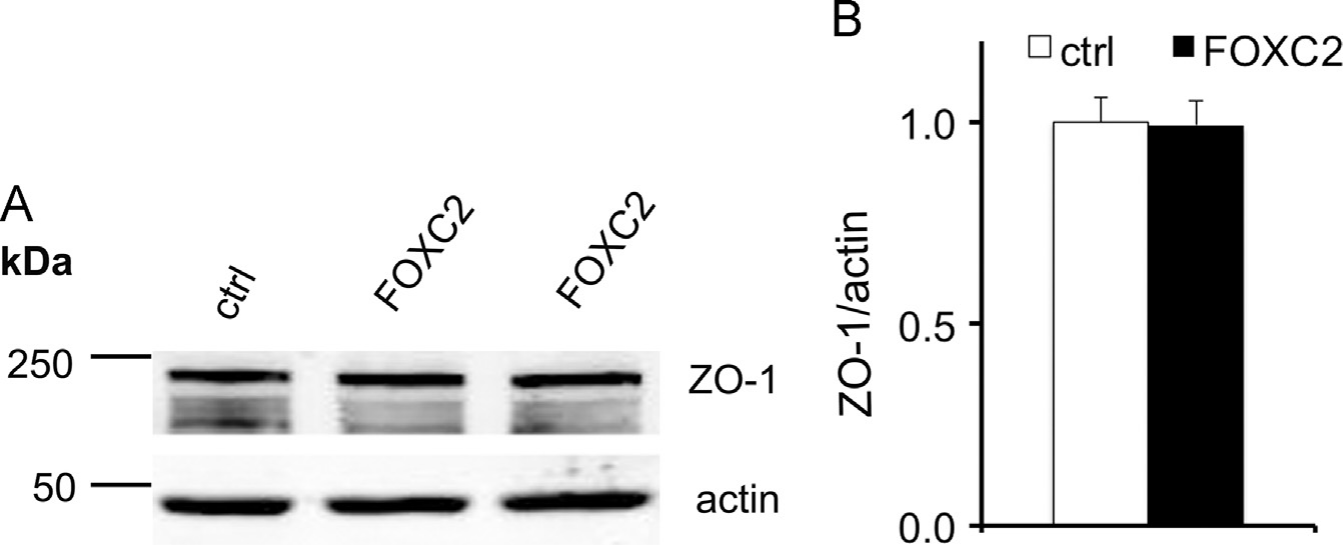
FOXC2 overexpression does not affect the expression level of ZO-1**.** (A) Immunoblotting for ZO-1 in differentiated podocytes transduced with control vector (ctrl) and FOXC2 construct. Actin is included as the loading control. (B) Quantification of the expression level of ZO-1 in three replicate blots and adjusted for actin reveals no difference in the expression level of ZO-1 in FOXC2 overexpressing podocytes compared to controls. Data are mean±SEM (*n*=3 experiments).

Quantitative Western blotting reveals that podocyte injury markers active beta-catenin, desmin and fibronectin show a trend of upregulation in the glomeruli of 40 weeks old obese Zucker rats compared to lean controls (Fig. 1A and B). Nephrin, the key protein of the interpodocyte slit diaphragm, shows a trend of downregulation in the glomeruli of obese rats (Fig. 1C and D), with the most albuminuric rats expressing least nephrin (Fig. 1E). Exposure of differentiated human podocytes to factors associated with obesity, insulin resistance and type 2 diabetes did not increase the expression of FOXC2 as observed by quantitative RT-PCR for tumor necrosis factor-α (TNF-α) and transforming growth factor β (TGF-β) (Fig. 2A and B), and by quantitative Western blotting for angiotensin II and a combination of glucose and palmitate (Fig. 2C-F). The data also show that overexpression of FOXC2 in differentiated human podocytes by lentiviral transduction does not change the ratio of filamentous (F) actin and globular (G) actin (Fig. 3A and B) or the expression level of the tight junction protein ZO-1 (Fig. 4A and B).

## 2 Experimental design, materials and methods

### 2.1 Animal model and preparation of glomerular lysates

Obese (fa/fa) and lean (fa/+) Zucker rats (Crl:ZUC-Leprfa) were obtained from Charles River Laboratories (Sulzfeld, Germany). Blood glucose values were measured from tail vein samples using OneTouch Ultra glucometer (Lifescan, Milpitas, CA). The urinary albumin to creatinine ratio was determined from spot urine samples. Albumin was measured with rat albumin ELISA kit (CellTrend, Luckenwalde, Germany) and creatinine using CREA plus enzymatic assay (Roche, Basel, Switzerland) and Roche clinical chemistry analyzer (Table 1). The experiments were approved by the National Animal Experiment Board. Glomerular fractions were isolated from 40 weeks old rat kidney cortices using the graded sieving method [2]. Cells were lysed in Nonidet P-40 (NP-40) lysis buffer (1% NP-40, 20 mM HEPES, pH 7.5, 150 mM NaCl) supplemented with 50 mM NaF, 1 mM Na_3_VO_4_ and 1× Complete Proteinase Inhibitor Cocktail (Roche, Basel, Switzerland) at 4 °C for 30 min. Detergent-insoluble material was removed by centrifugation (16,000*g* at 4 °C for 15 min).

### 2.2 Quantitative Western blotting

75 μg of glomerular lysates were separated on a 10% SDS-PAGE gel, transferred to PVDF-FL membranes (Millipore, Billerica, MA) and blocked with Odyssey blocking buffer (LI-COR, Lincoln, NE, USA) diluted 1:1 with phosphate buffered saline (PBS). The membranes were incubated with mouse anti-active β -catenin (Millipore, Darmstadt, Germany), mouse anti-desmin 37EH11 [3], rabbit anti-fibronectin (Abcam, Cambridge, UK), guinea pig anti-nephrin (Progen Biotechnik,Heidelberg, Germany), rabbit anti-ZO-1 IgG (Zymed Life Technologies, San Francisco, CA, USA), sheep anti-FOXC2 (R&D Systems, Minneapolis, MN, USA) and rabbit anti-actin (Abcam) IgGs, followed by Alexa Fluor 680 (Invitrogen) and IRDye 800 (LI-COR) anti-mouse, anti-rabbit, anti-guinea pig or anti-sheep IgGs. The signal was detected using an Odyssey Infrared Imager (LI-COR) and subsequently quantified using Odyssey software.

*Treatment of cultured human podocytes with obesity- and diabetes-associated factors*

Conditionally immortalized human podocytes (AB8/13) were cultured as described in Datta et al. [1]. Differentiated podocytes were serum starved for 12 h in medium supplemented with 1% fetal bovine serum (FBS) and independently treated with 10 ng/ml TNF-α (R&D Systems) for 2–24 h, 4 ng/ml TGF-β (R&D Systems) for 3 or 6 days, 1 μM angiotensin II (Sigma-Aldrich, St. Louis, MO, USA) for 24 h, or 20 mM glucose (Sigma-Aldrich) together with 200 μM palmitate (Sigma-Aldrich) for the last 7 days of the differentiation period. Sodium palmitate was conjugated with fatty acid-free low endotoxin BSA (Sigma-Aldrich) as described earlier [4]. Solvent only was used as a control for all treatments. Cells were either lysed and immunoblotted as described above or used for preparation of total RNA and quantitative RT-PCR as described below.

### 2.3 Total RNA preparation and quantitative RT-PCR

Total RNA was isolated, treated with DNase I and reverse transcribed into complementary DNA (cDNA). The quantitative PCR was performed using TaqMan gene expression assays (Applied Biosystems, Foster City, CA) for hFOXC2 (Assay ID: Hs00270951_s1) and hGAPDH (glyceraldehyde 3-phosphate dehydrogen- ase; Assay ID: Hs99999905_m1) as in [5]. Measurements were performed in triplicate using an iCyclerIQ^®^ (BIO-RAD, Hercules, CA, USA). The expression levels of FOXC2 mRNA were normalized to GAPDH using the comparative Ct method (DDCt).

### 2.4 Quantification of G-actin and F-actin

FOXC2 was lentivirally overexpressed in differentiated human podocytes as described in Datta et al. [1]. The G-actin and F-actin quantification in FOXC2 and control vector-transduced podocytes was performed as described in the G-actin/F-actin in vivo Assay Kit (Cytoskeleton Inc., Denver, CO, USA). Briefly, cells were lysed in a detergent-based lysis buffer that stabilizes and maintains the G- and F- forms of cellular actin. This was followed by a 100,000*g* centrifugation at 37 °C for 1 h that pellets the F-actin and leaves the G-actin in the supernatant. Samples of supernatant and pellet were separated by SDS-PAGE and actin was quantified by Western blotting using the rabbit polyclonal anti-actin antibody provided in the kit.

### 2.5 Statistical analysis

Results are presented as mean±SD. Statistical analysis was performed using Student׳s *t* test (Microsoft Excel, Redmond, WA).

## Figures and Tables

**Table 1:** Weight, blood glucose and urinary albumin to creatinine values of 40 weeks old lean and obese Zucker rats. The weights and blood glucose levels were not significantly different between the obese and lean rats, but the obese rats had significantly higher urine albumin to creatinine ratios.

**Zucker rats**	**Weight (g)**	**Blood glucose (mmol/l)**	**Urine albumin to creatinine (mg/mg)**
Obese	596±97.0 (*n*=6)	6.1±1.1 (*n*=6)	25.0±13.6⁎(*n*=3)
Lean	547±81.7 (*n*=6)	5.2±0.7 (*n*=6)	2.6±2.3(*n*= 6)

⁎ *p*<0.05.
